# The Distribution and Accessibility of Urban Parks in Beijing, China: Implications of Social Equity

**DOI:** 10.3390/ijerph16244894

**Published:** 2019-12-04

**Authors:** Shu Feng, Liding Chen, Ranhao Sun, Zhiqiang Feng, Junran Li, Muhammad Sadiq Khan, Yongcai Jing

**Affiliations:** 1 State Key Laboratory of Urban and Regional Ecology, Research Center for Eco-Environmental Sciences, Chinese Academy of Sciences, Beijing 100085, China; fs0212@126.com (S.F.); rhsun@rcees.ac.cn (R.S.); ksadiq259@gmail.com (M.S.K.); ycjing_st@rcees.ac.cn (Y.J.); 2 University of Chinese Academy of Sciences, Beijing 100049, China; 3 School of GeoSciences, The University of Edinburgh, Edinburgh EH8 9XP, UK; Zhiqiang.Feng@ed.ac.uk; 4 Department of Geosciences, The University of Tulsa, Tulsa, OK 74104, USA; junran-li@utulsa.edu

**Keywords:** urban public facility, inequality, Gini coefficient, Lorenz curve

## Abstract

As public service facilities, urban parks offer many benefits for daily life and social activities for residents. However, the accessibility of public parks to urban residents is often unevenly distributed in spaces that cannot be utilized fully. Here, we used the urban parks in Beijing, China as a case study and examined the relationship between urban park accessibility and population distribution at different administrative levels. Gini coefficient and Lorenz curve were used to evaluate the social equity of urban park accessibility, and the location quotient was used to identify the spatial difference between urban parks and resident population. The results of our study show that the urban park accessibility varies at district and subdistrict levels and that places with more urban parks usually have higher accessibility. Very importantly, the spatial equity is different from the social equity, a mismatch exists between the spatial distribution of urban parks and population, particularly for the elderly residents. These results generate valuable insights, as, in China and many developing countries, current urban public green space planning only uses the ratio of public green space to urban construction land and the per capita public green area.

## 1. Introduction

Green landscapes in metropolitan areas, such as urban parks, provide the city with various ecological, economic and social benefits, and are widely recognized as a critical component to the life quality of urban inhabitants [1,2]. Many studies have investigated the landscape patterns [3,4], ecological effects [5,6,7,8], health benefits [9,10], and accessibility [11,12,13] of urban parks. Additionally, urban parks provide opportunities for different types of leisure activities and play an important role in encouraging physical activities and social interactions among different communities, therefore reducing the stress and improving the physical and mental health of the residents [14,15].

The accessibility of urban parks is an important indicator of the park’s service and has also been viewed as a principal key to enhancing health and well-being [16,17]. There is evidence that access to nearby parks and open spaces is associated with physical and psychological health, such as mood change, reduced anxiety, and childhood obesity [18]. These benefits may be particularly important for people with reduced mobility and cognitive function [19]. For example, Maas et al. (2009) [20] argued that the elderly (aged 65 +) may feel more social support and less lonely when living close to green spaces, and other studies showed that access to parks may increase their longevity [21,22]. 

In recent years, the accessibility of urban parks has been increasingly considered in the contexts of social equity and environmental justice [23,24,25]. For example, in China, the implementation of social justice and ecologically-minded civilization requires that residents can access the public park resources easily and equally. In the United States, the National Recreation and Park Association (2011) has undertaken a comprehensive review and found park provision inequity. Additionally, the Millennium Ecosystem Assessment indicated that urban parks can enhance social equity by creating and maintaining public spaces and natural environments for social interactions [26,27]. The reasonable spatial distribution of urban parks is therefore of great significance to realize and equal public services and social equity, involving different socioeconomic status or ethnic/ethnic composition. 

Research on urban parks mainly focuses on rational allocation, and the evaluation of the accessibility and fairness of urban parks is an important part. Based on the geographic information system (GIS), the spatial equality of urban parks is analyzed through accessibility, and the social equity of urban parks is evaluated by considering the needs and spatial distribution of different social groups [28,29]. In China, the per capita park green area ratio is a common indicator used in urban park planning, and the development of urban parks often emphasizes size and quantity, paying less attention to the match between the spatial distribution of urban parks and user needs [30]. Previous studies mainly focused on evaluating the availability and accessibility of urban parks in various aspects [31,32]. In recent years, a few studies on urban parks have gradually turned to the match-up between urban parks and population distribution, and started to consider the needs of specific groups, reflecting the concept of equity and justice [33,34]. However, studies on social equity in urban parks are still insufficient, especially for vulnerable groups such as the elderly. 

In aiming to fill the aforementioned research gap in the social equity of urban parks, we selected the area inside the 5th ring-road of Beijing as the study area, and used the Gini coefficient and the Lorenz curve to evaluate the social fairness of urban park accessibility based on the concept of social equity. We further used the location quotient method to evaluate the spatial matching between urban parks and the population distribution. The objectives of this study are to: (1) analyze the social equity of urban park accessibility and the spatial distribution patterns of urban parks; (2) evaluate the relationships between urban park accessibility and population distribution for all residents and elderly residents; and (3) find implications of social equity for urban park planning. 

## 2. Materials and Methods

### 2.1. Study Area

**Figure 1:**
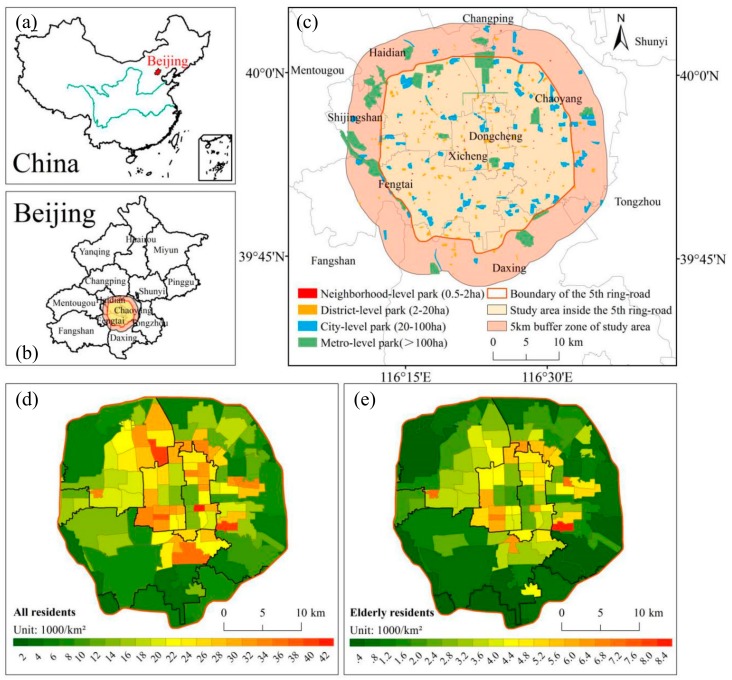
Location of Beijing in China (**a**), the distribution of the 16 districts (**b**), various levels of urban parks (**c**), population density of all residents at subdistrict-level (**d**), and population density of elderly residents (residents aged 60 and over) at subdistrict-level (**e**) in the study area.

Beijing (39°26′–41°03′ N, 115°25′–117°30′ E), located in northern China, is the national center of politics, culture, international exchanges, and technological innovation (Figure 1a). Beijing is divided into 16 districts and numerous subdistricts and its urbanization is developed in a circular pattern from the downtown to the suburb (Figure 1b). This study focuses on the highly urbanized region located within the 5th ring-road of Beijing, covering an area of 667 km^2^. The study area includes the whole or part of seven districts and 119 smaller subdistricts. By the end of 2017, about 48% of the total population and more than half of the elderly in Beijing lived in this area. Although some large green spaces are located outside the 5th ring-road, they also become critical public space for urban residents inside the 5th ring-road if they are easily accessible. Thus, we set up a buffer zone of 5 km around the 5th ring-road to include those green parks in this study (Figure 1c). 

From 1989 to 2009, the area of urban green space in Beijing decreased 15%. The loss of green space mainly occurred in the urban fringe region between the 4th and 5th ring-road [35]. Since the beginning of the 21st century, comprehensive projects have been initiated to enlarge urban green spaces and establish a green corridor network. These projects have dramatically improved the city’s landscape and provided more recreational places for local people. The latest Beijing Master Plan (2016–2035) stipulates specifically that the government will continue to strengthen urban forest restoration and create a comfortable living environment for the citizens. 

### 2.2. Urban Park Service Radius

We used the latest Google satellite aerial maps (https://www.google.com/earth/), as well as the Gaode electronic maps (https://www.amap.com/), to create a vector map of each urban park. The Natural England’s Accessible Natural Greenspace Guidance, the Accessible Natural Greenspace Standards (ANGSt) [36], recommend that “everyone, wherever they live, should have an accessible natural greenspace of at least 2 ha in size, no more than 300 m from home; at least one accessible 20 ha site within 2000 m of home; one accessible 100 ha site within 5000 m of home……”. In China, some urban planning documents, for example, the Evaluation Standard for urban landscaping and greening (GB/T 50563-2010), the Code for the Design of Public Park (GB 51192-2016), and the Regulatory Plan for the Central City of Beijing (2004–2020), recommend that urban parks within the built-up area should be no less than 0.5 ha, and residents should have access to public green space in the vicinity of 500 m. In this study, we chose the ANGSt and considered the actual situation in China, and determined that 0.5 ha is the minimum size of an urban park. The urban parks larger than 0.5 ha were further classified into four categories according to their size: neighborhood-level park (0.5–2 ha); district-level park (2–20 ha); city-level park (20–100 ha); and metro-level park (> 100 ha). Liu and Li (2010) [37] argued that the service radius of a park is proportional to its size. In this study, we designated the service radius of 500 m, 1000 m, 2000 m and 5000 m, respectively, for the four categories of parks defined above [34,36]. 

### 2.3. Urban Park Accessibility

The accessibility of the urban parks was analyzed at the district-level and the subdistrict-level. We used a green space accessibility (GSA) index proposed by Fan (2016) [38] to quantify the linkage between the resident’s location and urban parks in the study area. Compared with other methods, this index can better reflect a location’s access to available green spaces at different levels and how well a location can access different types of urban parks in a city. The urban park vector layer was converted to raster with a resolution of 10 m, and the urban park accessibility was calculated by GIS software as:

(1) $${\mathrm{GSA}}_{i}=\left\{\begin{array}{c} 0\\ 1-\frac{{\mathrm{dist}}_{i}}{D_{i}}\;\;({\mathrm{dist}}_{i}<D_{i}\\ 1 \end{array}\right.)$$

where GSA_i_ is the accessibility of the pixel i to the i^th^ park and it varies from 0 to 1, dist_i_ is the shortest distance (the straight-line distance) from the pixel to the edge of the i^th^ park, D_i_ is the service radius of the park i. GSA_i_ to ith park is equal to 1 if the pixel is located in the green space, 0 if the shortest distance is beyond D_i_, or a value between 0 and 1 if the shortest distance is within D_i_.

For neighborhood-level parks, GSA = GSA_Ni_, dist_i_ = dist_Ni_, D_i_ = D_Ni_ = 500; 

For district-level parks, GSA = GSA_Di_, dist_i_ = dist_Di_, D_i_ = D_Di_ = 1000; 

For city-level parks, GSA = GSA_Ci_, dist_i_ = dist_Ci_, D_i_ = D_Ci_ = 2000; 

For metro-level parks, GSA = GSA_Mi_, dist_i_ = dist_Mi_, D_i_ = D_Mi_ = 5000.

The accessibility (GSA) of a pixel is defined as the summary of accessibility to all nearby parks (i = 1,…n), 

(2) $$\mathrm{GSA}=\overset{n}{\underset{i=1}{\sum }}({\mathrm{GSA}}_{\mathrm{Ni}}+{\mathrm{GSA}}_{\mathrm{Di}}+{\mathrm{GSA}}_{\mathrm{Ci}}+{\mathrm{GSA}}_{\mathrm{Mi}})$$

GSA ranges from 0 to 4, and it refers to the accessibility evaluation of all the urban parks. The average GSA of a district or subdistrict was calculated based on all pixels in that area. 

### 2.4. Social Equity Evaluation

The Lorenz curve, Gini coefficient, and Location quotient were used to develop an index to examine the social equity of urban parks.

#### 2.4.1. Lorenz Curve and Gini Coefficient

The Lorenz curve is a widely used graphical representation of income or wealth inequality, and it shows the relationship between population (%) and income wealth (%) [39,40,41]. In our study, the Lorenz curve was used to indicate the relationship between the accessibility of urban park resources and the resident population. All spatial units in the study area were ranked according to per capita urban park resources accessibility from low to high, and the proportion of urban park resources shared by the resident population in each interval of 10% was also calculated. The Gini coefficient, which was developed based upon the Lorenz curve, is calculated using the following formula [42]:

(3) $$G=1-\overset{n}{\underset{k=1}{\sum }}\left(P_{k}-P_{k-1}\right)\left(R_{k}+R_{k-1}\right)$$

where P_k_ is the accumulative percentage of resident population, R_k_ is the accumulative percentage of urban park resource accessibility. The Gini coefficient ranges from 0 to 1, and 0 reflects complete equality and 1 indicates complete inequality. A low Gini coefficient indicates a more equal distribution of the urban park resources, and we considered 0.4 as a warning line of inequality [43]. Population data was collected from the sixth census data of China. The population density was calculated based on the people dwelled in each subdistrict (Figure 1d,e).

#### 2.4.2. Location Quotient (LQ)

The location quotient is an indicator that measures the specialization level of a single industry in a particular region and reflects the industrial concentration. Although this index has been traditionally used in regional economics [44,45], Tang and Gu (2016) [33] successfully used this index to analyze the spatial pattern of social equity performance. In this study, we set the location quotient of each spatial unit as the ratio of the urban park resources shared by the resident population in the space unit compared to the average urban park resources shared per capita by the resident population in the whole study area. The calculation formula is:

(4) $${\mathrm{LQ}}_{i}=\left(T_{i}/P_{i}\right)/\left(T/P\right)$$

where, LQ_i_ is the location quotient of the space unit i, T_i_ and T are the urban park resource in space unit i and the total amount of urban park resources in the study area, respectively, and P_i_ and P are the resident population in space unit i and the total resident population in the study area, separately. A location quotient > 1 indicates that the per capita share of the urban park resources in the space unit is higher than the average of the study area, whereas a location quotient < 1 suggests that the per capita share of the urban park resources in the space unit is lower than the average of the study area. 

## 3. Results

### 3.1. Spatial Distribution of Urban Parks

A total of 333 urban parks were identified in the study area (Figure 1, Table 1). Among these urban parks, 152 are district-level parks, which account for nearly 46% of all parks, while their area only accounts for 10% of the total area of the urban parks in the study area. Only 22 parks are classified as metro-level parks, which have an average area of 287.8 ha and account for 54% of the area of all urban parks in the study area in Beijing. 

**Figure 2:**
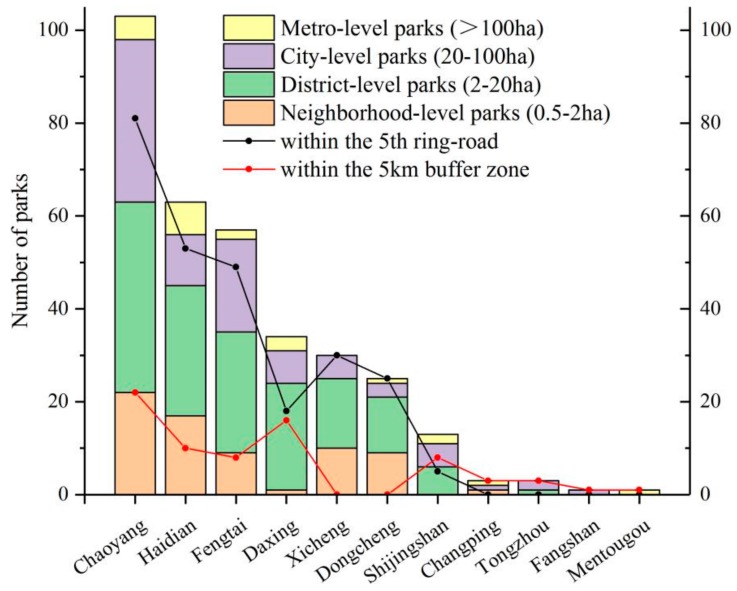
The distribution of different level urban parks in Beijing, China.

Of the urban parks, 261 are located in the study area inside the 5th ring-road, and 72 are in the 5 km buffer zone of the study area. The distribution of different levels of urban park showed that 103 urban parks are located in Chaoyang, followed by Haidian and Fengtai with 63 and 57, respectively (Figure 2). The districts of Daxing, Dongcheng and Xicheng each has about 30 parks. By contrast, Shijingshan only has a few parks. Among four types of urban parks, the neighborhood-level parks are mainly located in Chaoyang, Haidian, Xicheng, Dongcheng, and Fengtai. Both district-level and city-level parks are located in almost all districts, but the metro-level parks are primarily found in Chaoyang, Haidian and Daxing, followed by Fengtai and Shijingshan. 

### 3.2. Urban Park Accessibility

**Figure 3:**
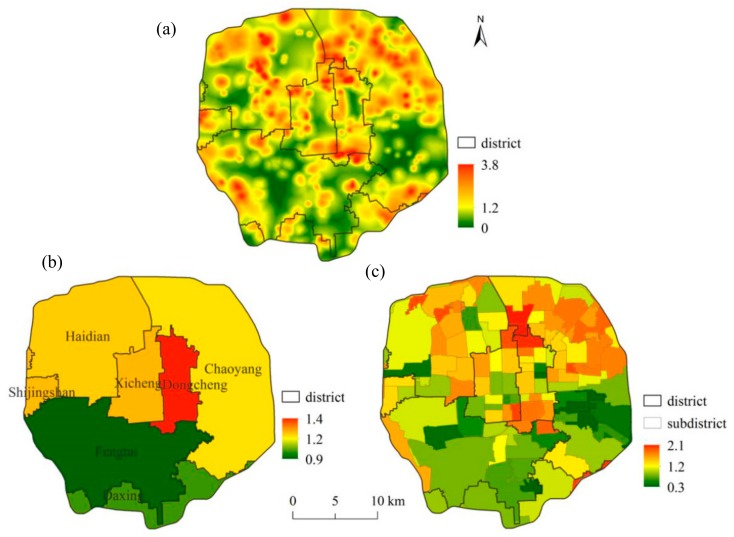
Spatial distributions of the urban park accessibility (green space accessibility; GSA) at various levels, (**a**) the entire study area, (**b**) the district-level, (**c**) the subdistrict-level.

The average urban park accessibility (GSA) within the 5th ring-road of Beijing is 1.2 (Figure 3a). About 93% of the study area has the GSA varying between 0 and 2, and only 0.1% of the area has a GSA > 3 (Table 2). Areas with high GSA are primarily distributed in central Haidian, northern Chaoyang, the Dongcheng, Xicheng and Chaoyang intersection area, and the Dongcheng and Fengtai intersection area. However, 12.7% of the area still has a GSA < 0.5, where only a few urban parks are located.

At the district-level, GSAs of Dongcheng, Xicheng, Chaoyang, Haidian, and Shijingshan are higher than the average of the study area, and the GSA of the central Dongcheng is the highest (Figure 3b). The values of GSA in Daxing and Fengtai are lower than the average of the study area, of which Fengtai has the lowest value of 0.9. 

At the subdistrict-level, the northern and northwestern regions of the study area have higher GSA (Figure 3c). A total of 69 subdistricts have GSAs above the average of 1.2, accounting for 60% of the total 119 subdistricts in the study area. Of all the subdistricts, about 27.7% of them have GSA ranging between 0 and 1, and 71.4% of them vary between 1 and 2 (Table 2). 

### 3.3. Social Equity of Urban Park Accessibility

**Figure 4:**
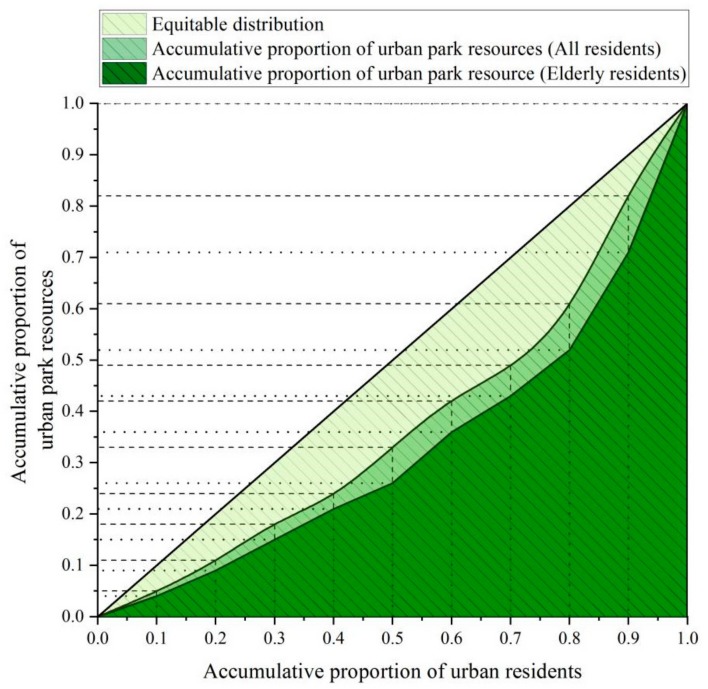
Lorenz Curve for the distribution of urban park resources in Beijing, China.

The Gini coefficient of urban park resource allocation for all residents within the 5th ring-road area is 0.25, and the Gini coefficient for elderly residents is about 0.35, which is close to the warning line of 0.4. The Lorenz curve further shows that the urban park resources are more unequally distributed among elderly residents than all residents (Figure 4). It shows that the top 10% of all residents can use 18% of the total urban park resources, while the same proportion of the elderly can use about 29% of the resources. In contrast, for the all residents of the bottom 10%, they can access only 5% of the total urban park resources, and the same proportion of the elderly can only access 4% of the resources.

### 3.4. Spatial Patterns of Social Equity

**Figure 5:**
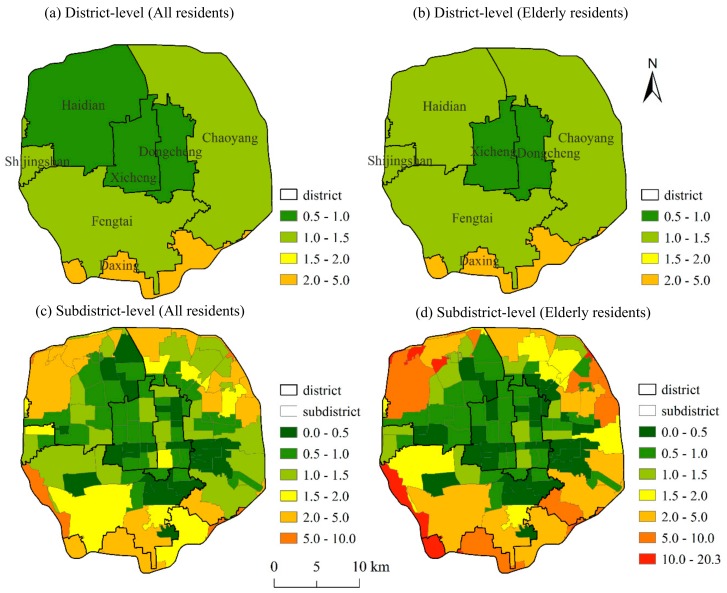
The distribution of location quotients (LQs) for all residents and elderly residents at the district (**a**,**b**) and subdistrict levels (**c**,**d**) in Beijing, China.

At the district-level, the LQs in Xicheng and Dongcheng are less than 1, indicating that the per capita urban park resources are lower than the overall level. The LQs in the eastern and southern regions are higher than the rest (Figure 5a). At the subdistrict-level, the LQs in the central areas are generally lower than those in the peripheral areas (Figure 5c). Nearly 52% of the subdistricts have LQs < 1, and around 20% of the subdistricts have LQs < 0.50 (Table 3). The spatial units with LQs > 5 are mainly located in the area with extensive public green spaces, emerging industrial parks, and newly-built residential areas with low resident density.

For the elderly residents, there are more subdistricts with very low and very high LQs than for residents overall (Figure 5b,d). For more than half of the subdistricts (56.3%), the LQs are less than 1 (Table 3), and for 13.4% of subdistricts LQs are greater than 5.

## 4. Discussion

Numerous studies have examined the accessibility and usability of urban parks in developed and developing countries [38,46,47,48,49,50]. Many studies have demonstrated that the distribution of green spaces is often related to the geographical locations and the development history in the urban area [51,52,53,54,55]. These studies, however, failed to link the characteristics of the parks and the urban residents. In our study, we analyzed the spatial patterns of both space–based and population–based urban park resources, and attempted to reveal the internal relationships between the urban park accessibility and the resident population at district and subdistrict levels. We paid close attentions to a special age group, the elderly residents, in our analyses, with the consideration that the accessibility of urban parks is of particular significance to their health and life quality. 

### 4.1. Population Distribution and Urban Park Accessibility

Our study confirmed that the neighborhood–level parks are mainly distributed in central Beijing, including the districts of Chaoyang, Haidian, Xicheng, and Dongcheng, while the metro-level parks are mainly distributed on the border of the study area. The accessibility of these large, metro-level parks, however, does not match with the distribution of the population who are in need of the park resources. This study showed that the population density of Beijing, especially the elderly people, increases from the periphery to the centre of the city, while the LQs in the central areas are generally lower than those in the peripheral areas. The results demonstrate that the characteristics of the population may be a direct factor that leads to the social inequality of park resources in Beijing. Therefore, understanding social equity relies on knowing who or what group the majority of people deem worthy [56].

In recent years, the structure, distribution, and demands of Beijing’s population have changed substantially, posing new challenges to the public services including the urban parks [57,58]. First, the proportion of elderly residents has increased steadily from 12.5% in 2010 to 16.5% at the end of 2017. Second, the population has gradually decentralized from the central Beijing area (e.g., Dongcheng, Xicheng, Chaoyang etc.) to the suburbs. Third, the continuous increase of income has made the demand for public green space services more diversified and personalized. For example, high–income people may prefer parks with grass and flexible pavement and expect less walking time [59], while low-income people such as retired elderly, children and housewives often participate in some group entertainments and prefer the public leisure space in the park. All these demographic changes will greatly alter the population’s demands, and understanding these will help the planning, construction, or adjustment of urban parks to achieve social equity.

### 4.2. Implications for Urban Park Planning

The spatial configuration of urban parks is a typical user-related issue, which means that the demands of users need to be analyzed, including the location of the demanders and how much demand they have [30,34,60]. This research focuses on evaluating the spatial allocation of urban parks from the perspective of social equity, with particular attention to the matching relationship between the spatial distribution of urban parks (supply–side) and the resident population (demand–side) in the same area. 

The results of our study show that indicators currently used in urban public green space planning, that is, the ratio of public green space to urban construction land and the per capita public green area, are not able to reflect the social equity of the urban park resources, that is, the accessibility of the urban parks to different aging groups. Although other studies have paid attention to the accessibility and usability of parks or greenspaces, most of them considered the spatial accessibility, but not the social equity, of park service [13,38,61]. However, a few studies have confirmed that there were spatial inequality and spatial disparities based on the supply–demand relations of urban parks [62,63], and previous consideration of urban park distributions did not emphasise the importance of the demands of the elderly and children [34]. Our results further confirmed that spatial equity was significantly different from social equity, and a spatial mismatch still exists between the distribution of urban parks and population, particularly for the elderly residents. These findings suggest that the pattern of place–based equity is different from population–based social equity, and the high urban park accessibility does not equate to high social equity. 

Reducing the inequality in access to public services is considered an essential way to achieve sustainable development and to improve the livelihoods of all populations [64]. The results of our study suggest that the equity of the park service not only should cover as many areas as possible but also should consider the needs and preferences of different groups. There should be no circumstances where some areas are served by many parks, while others do not have any around them [28,65]. Identifying the mismatch between supply and demand, especially for different socioeconomic status or ethnic/ethnic composition in cities, will greatly help urban planners optimize the allocation of urban parks to provide equal public services and social equity. 

## 5. Conclusions

The results of our study highlight the fact that urban park accessibility varies at different spatial scales and there is a mismatch between the distribution of urban parks and urban residents. The Gini coefficient of urban park resource allocation for elderly residents is higher than that of residents overall, and the mismatch exists between the spatial distribution of urban parks and population, especially for the elderly residents, indicating that the optimal allocation of urban park resources should pay particular attention to the elderly’ demands. This study provides useful insights into the development of targeted urban public facilities planning strategies to address social inequities.

A few issues should be addressed in the future studies. First, many other factors affecting the accessibility of the urban parks, such as the quality of the park (facilities distribution, comfort, safety, artistic value, etc.), the real willingness of residents’ travel preferences and travel modes [66,67], are not considered in the current study. Second, the method of assessing the GSA based on physical linear distance in this paper may only reflect one aspect of the accessibility, therefore a more comprehensive evaluation system needs to be developed. Third, although some studies have used the Gini coefficient to assess social equity of urban green space and public transportation [33,42,50], the allocation of public green space resources is not the same as income distribution. Whether the Gini coefficient can be used to accurately judge an urban park’s spatial distribution still needs to be further explored by a larger number of empirical studies. Finally, in addition to the elderly population, low-income people are also an important part of China’s human population structure. Understanding their spatial distribution and demand preferences will no doubt help address specific inequities, and achieve social equity and justice for the city.

## Figures and Tables

**Table 1:** The number and area distribution of the urban parks in Beijing, China.

Type of Parks	Number		Area		
	Count	Percentage (%)	Area (ha)	Percentage (%)	Average Area (ha)
Neighborhood-level (0.5–2ha)	69	20.7	72.5	0.6	1.1
District-level (2–20ha)	152	45.7	1163.9	10.0	7.7
City-level (20–100ha)	90	27.0	4067.9	35.0	45.2
Metro-level (>100ha)	22	6.6	6330.8	54.4	287.8
Total	333	100.0	11635.0	100.0	—

**Table 2:** The distribution of different categories of GSA in Beijing, China.

Range of GSA	Entire Study Area	Subdistrict-Level	
	Percentage (%)	Number of Subdistricts	Percentage (%)
0–0.5	12.7	3	2.5
0.5–1.0	27.6	30	25.2
1.0–1.5	32.0	49	41.2
1.5–2.0	20.8	36	30.3
2.0–3.0	6.8	1	0.8
>3.00	0.1	0	0.0

**Table 3:** The characteristics of the urban park location quotients (LQs) in Beijing, China.

LQ	All Residents		Elderly Residents	
	Number of Subdistricts	Percentage (%)	Number of Subdistricts	Percentage (%)
0–0.5	24	20.2	33	27.7
0.5–1.0	38	31.9	34	28.6
1.0–1.5	25	21.0	15	12.6
1.5–2.0	10	8.4	8	6.7
2.0–5.0	16	13.4	13	10.9
5.0–10.0	6	5.0	10	8.4
>10.0	0	0.0	6	5.0
